# Transcriptome of Protoplasts Reprogrammed into Stem Cells in *Physcomitrella patens*

**DOI:** 10.1371/journal.pone.0035961

**Published:** 2012-04-24

**Authors:** Lihong Xiao, Liechi Zhang, Ge Yang, Honglin Zhu, Yikun He

**Affiliations:** 1 College of Life Science, Capital Normal University, Beijing, People's Republic of China; 2 Beijing Computing Center, Beijing, People's Republic of China; University of North Carolina at Charlotte, United States of America

## Abstract

**Background:**

Differentiated plant cells can retain the capacity to be reprogrammed into pluripotent stem cells during regeneration. This capacity is associated with both cell cycle reactivation and acquisition of specific cellular characters. However, the molecular mechanisms underlying the reprogramming of protoplasts into stem cells remain largely unknown. Protoplasts of the moss *Physcomitrella patens* easily regenerate into protonema and therefore provide an ideal system to explore how differentiated cells can be reprogrammed to produce stem cells.

**Principal findings:**

We obtained genome-wide digital gene expression tag profiles within the first three days of *P. patens* protoplast reprogramming. At four time-points during protoplast reprogramming, the transcript levels of 4827 genes changed more than four-fold and their expression correlated with the reprogramming phase. Gene ontology (GO) and pathway enrichment analysis of differentially expressed genes (DEGs) identified a set of significantly enriched GO terms and pathways, most of which were associated with photosynthesis, protein synthesis and stress responses. DEGs were grouped into six clusters that showed specific expression patterns using a K-means clustering algorithm. An investigation of function and expression patterns of genes identified a number of key candidate genes and pathways in early stages of protoplast reprogramming, which provided important clues to reveal the molecular mechanisms responsible for protoplast reprogramming.

**Conclusions:**

We identified genes that show highly dynamic changes in expression during protoplast reprogramming into stem cells in *P. patens*. These genes are potential targets for further functional characterization and should be valuable for exploration of the mechanisms of stem cell reprogramming. In particular, our data provides evidence that protoplasts of *P. patens* are an ideal model system for elucidation of the molecular mechanisms underlying differentiated plant cell reprogramming.

## Introduction

Cell reprogramming is an important biological phenomenon whereby cells regress from a specialized, differentiated state to a simple, undifferentiated cell type reminiscent of stem cells with the capability for both self-renewal and to give rise to most other cell types in multicellular organisms [1], [2]. Asymmetric division of a stem cell generates two different daughter cells: a self-renewed stem cell daughter that retains the stem cell's pluripotent characteristics, and a differentiated non-stem cell daughter [3]. Stem cells in plant shoot and root meristems are maintained throughout the life of plants and produce somatic daughter cells that make up the body of plants [4]. Commonly, specialized cells are formed by a one-way process as a fertilized egg develops into an adult, and the cells become increasingly, and normally irreversibly, committed to their fate. However, certain experimental procedures can reverse the cell differentiation process and cause cells to acquire a new fate by reprogramming, a term that describes a switch in nuclear gene expression in one kind of cell to induce it to differentiate into a different cell type. A distinctive feature of cell reprogramming is the withdrawal from a given differentiated state into a stem cell-like state that confers pluripotentiality, a process that precedes the switch in cell fate [5]. This process underlies the totipotency, regeneration and formation of new stem cells. Elucidation of how cell reprogramming takes place is important to help us understand the mechanisms by which cell division and differentiation occur.

Reprogramming of a differentiated cell to become a pluripotent stem cell is frequently observed in plants and is more easily induced in plants than in animals. Differentiated plant cells, in contrast to those of animals, hold multiple developmental potentialities during development and retain a plasticity that enables dedifferentiation [6]. However, the genetic and molecular bases of this difference between plant and animal cells are mostly unknown.

Recently, artificial expression of two transcription factors, Oct4 and Sox2, together with other factors made it possible to reprogram differentiated somatic cells into pluripotent stem cells in mice and humans [7]. The study of cellular reprogramming in animals is limited because of the lack of a suitable, convenient experimental system [8]. Plant protoplasts (plant cells devoid of cell walls) provide an outstanding experimental tool for the study of the biochemical and molecular basis of cellular reprogramming into stem cells [6], [9], [10].

Application of phytohormones, such as auxins and cytokinins, stimulate protoplasts from different tissues to reenter the cell cycle, proliferate, and undergo regenerative processes to give rise to new plantlets [11]–[13]. Using a plant protoplast system, Zhao et al. [9] demonstrated that protoplasts can be isolated easily from fully differentiated, non-dividing mesophyll cells of tobacco leaves, and reenter the cell cycle and proliferate following treatment with auxin and cytokinin. These authors also found that the reprogramming of tobacco mesophyll cells proceeds by two functionally distinct phases of chromatin decondensation: the first is a transitory phase that confers competence for a switch in cell fate followed, under appropriate hormonal conditions, by a second phase that represents commitment to the mitotic cycle. Subsequent studies of protoplast reprogramming concentrated on the relationship between transcription and the structure of chromatin that is involved in heterochromatin decondensation and histone modification [9], [10], [14], [15].

Compared to vascular plants, bryophytes and ferns have a single stem cell in the protonema tip and leafy shoot apex, lack a stem cell niche and organization center, and possess an accessible haploid and relatively simple structure [16], [17], and thus represent a simpler experimental system. Although they diverged hundreds of millions of years ago, bryophytes share similar fundamental genetic and physiological features with seed plants [18] and are phylogenetically intermediate between algae and seed plants [19]. Among bryophytes, the moss *Physcomitrella patens* has emerged recently as the bryophyte model of choice for studies of development, genetics and stress responses [20]–[23].

The *P. patens* apical stem cell system has received much attention following its description in 2007 (). Specifically, differentiated cells from any part of the gametophyte or sporophyte, including an excised leaf, protonemal cells or freshly isolated protoplasts, can be easily reprogrammed into protonemal apical stem cells without exogenous phytohormone treatment within a few days [24]. A recent study on reprogramming of excised leaf cells indicated that a cell cycle protein kinase A (CDKA) regulates cell division and acquisition of new cell characteristics in the reprogramming of differentiated cells to become stem cells in plants [25]. However, the factors that coordinate cell cycle reactivation with acquisition of other cellular characteristics during protoplast reprogramming into stem cells have not been determined.

In this study, we utilized the *P. patens* protoplast system to explore the mechanisms and key candidate regulators involved in stem cell reprogramming. The objectives of the present study were to: (1) characterize changes in gene expression associated with protoplast reprogramming, (2) investigate the molecular mechanisms responsible for protoplast reprogramming, and (3) identify candidate genes and key factors involved in protoplast reprogramming into stem cells. By combining the *P. patens* protoplast system with a digital gene expression tag profiling (DGEP) strategy, we obtained spatiotemporal-specific gene regulation models for protoplast reprogramming. These results provided a comprehensive catalogue of gene expression changes during protoplast reprogramming, from which potentially key regulatory factors can be mined.

## Results

### Morphogenesis of protoplasts reprogrammed into stem cells

**Figure 1:**
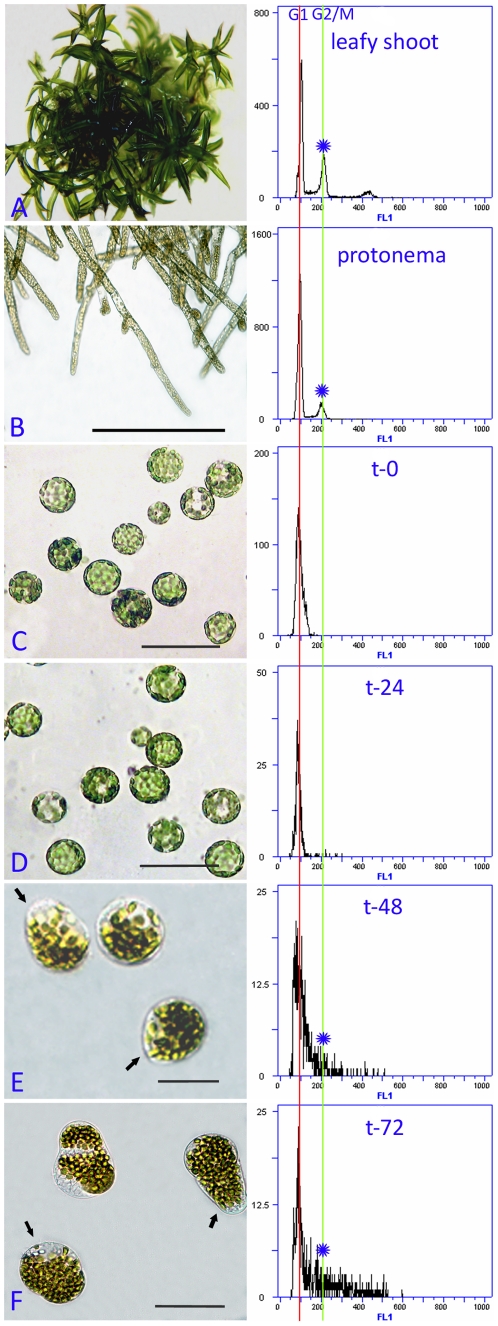
Morphology and evaluation of cell cycle by flow cytometry. (A) Two-month-old leafy shoots. (B) Six-day-old protonemata. (C) Freshly prepared protoplast. (D) Cells cultured for 24 hours. (E) Cells cultured for 48 hours. (F) Cells cultured for 72 hours. The stem cells are indicated by arrows in each stage. The S phase of cell cycle is represented by the area between the two red broken lines, and the G2/M phase is indicated by a blue star in each stage.

To investigate how protoplasts were reprogrammed into stem cells, six-day-old subcultured *P. patens* protonemata (Fig. 1B) were used to establish an efficient and reproducible ‘protoplast system’ in *P. patens*. When protonemal tissue was treated to form protoplasts, the cells changed their nuclear program and lost their differentiated state (Fig. 1C). Protoplasts freshly isolated from protonemata were round and green because of the presence of chloroplasts. Upon culture in the dark, more than 90% of the protoplasts developed a new cell wall within 24 h of culture (Fig. 1D). The new polar axes were re-established within 48 h and up to 87% of the cells were pyriform (Fig. 1E), which indicated that protoplasts are reprogrammed to acquire at least some stem cell characteristics before mitosis. After 72 h, 85% of the originally plated protoplasts divided asymmetrically and yielded chloronema that contained two to three cells, of which the apical cell was a self-renewing stem cell and other cell(s) were differentiated non-stem cell(s) (Fig. 1F). Subsequently, the cultures were transferred to a propagation and regeneration medium (BCDAG medium) for formation of protonema clones, the apical cells of which were stem cells. Using this protoplast system, we followed alterations in the cell cycle and DGEP analysis at subsequent time intervals up to 72 h after protoplast isolation.

### Protoplast reprogramming into stem cells occurs within 48 hours

To evaluate the time course of protoplast reprogramming into stem cells, we measured the DNA content of nuclei isolated from freshly prepared (t-0) protoplasts and protoplast-derived cells cultured for 24, 48 or 72 hours (t-24, t-48 and t-72), respectively, by flow cytometry (FCM). In order to ascertain the cell cycle phase of protoplasts, we used DAPI-stained nuclei from two-month-old leafy shoots and six-day-old protonemata of *P. patens* as a control (Fig. 1A–B). Under the experimental conditions, six-day-old protonemata mainly contained chloronema cells and rarely caulonemal cells (Fig. 1B). Nuclei from two-month-old leafy shoots exhibited three peaks that corresponded to G1- and G2-phase as well as polyploidization, which may be the results of endoduplication (Fig. 1A). Six-day-old *P. patens* protonemal nuclei displayed double peaks that corresponded to G1- and G2/M-phase DNA content, respectively (Fig. 1B). Comparison of the FCM results for the controls, we can speculate that most nuclei were in the G1 phase of the cell cycle with a only small number of cells in the S and G2/M phases, which differed from previous reports that chloronema cells were arrested in the G2 phase [26], [27].

Comparison of the flow cytometry histograms of DAPI-stained nuclei isolated from t-0 protoplasts and protoplast-derived cells (t-24, t-48 and t-72 cells) with nuclei prepared from two-month-old leafy shoots and six-day-old protonemata indicated that nuclei prepared from both t-0 protoplasts (Fig. 1C; G1 nuclei) and t-24 cells (Fig. 1D; G1 nuclei) reproducibly showed a single peak. These results indicated that t-0 protoplasts and t-24 protoplast-derived cells underwent synchronization. The t-24 protoplast cultures were highly synchronized and almost all cells were in the G1 phase. Few t-48 cells showed an increase in fluorescence intensity compared with t-24 protoplasts (Fig. 1E). A portion of the t-48 cells were in the S phase and the other cells were in the G2/M phase (Fig. 1). This result implied that the protoplasts had been reprogrammed and re-entered the cell cycle. Features of t-72 cells were similar to those of t-48 cells (Fig. 1). From integration of FCM analysis with morphogenetic observations during protoplast culture, we concluded that protoplast reprogramming into stem cells occurred within 48 h.

### Tag identification and quantification and depth of sequencing

To obtain global patterns of gene expression during protoplast reprogramming, RNA extracted from fresh protoplasts and cells cultured for 24, 48 and 72 h was used for DGEP analyses. More than 3.2 million raw tags (Table 1) were sequenced using the cDNA library derived from fresh protoplasts and cells cultured for 24, 48 and 72 h (Table 1). Custom Perl scripts were used for adaptor trimming and read parsing. Before mapping these tag sequences to the reference sequence, low-quality tags (tags containing ‘N’ and adaptor sequences) were filtered. To increase the robustness of the approach, single-copy tags in the four libraries were excluded [28]. The distribution of distinct clean tag counts over different tag abundance categories showed very similar tendencies for all four libraries (Fig. S1) [29], [30]. Common and specific tags within and among samples are shown in Fig. S1.

**Table 1:** Major characteristics of DGEP libraries and mapping information.

Sample	Raw	Trim adaptor	Drop low quality	Drop CopyNum = 1 (clean tags)	Distinct clean tags	Mapped tags	Mapping rate
0 h	3503924	3420205	3420205	3314401	135590	113281	83.55%
24 h	3202434	3061865	3023002	2743030	207118	135265	65.31%
48 h	3719941	3173209	3166328	2986395	92100	135265	75.32
72 h	7762517	3487600	3474314	3373054	98148	72965	74.34%

Trim adaptor: adaptor tags were filtered.

Drop low quality: low quality tags were filtered.

Drop CopyNum = 1: tags of copy number = 1 were filtered.

Distinct clean tags: different kinds of clean tags.

Saturation of the library was determined by identification of unique tags. Sequencing reached saturation when no new unique tags were detected. The results shown in Fig. S2 indicated that all four sampling libraries were sequenced to saturation, and thus a full representation of the transcripts in the experimental conditions was obtained. In the four libraries fewer unique tags were identified as the number of sequencing tags increased, and reached a plateau shortly after 2 million tags were sequenced and a negligible increase in the number of genes detected in the four libraries was observed.

### Mapping of short reads to the reference genome and detection of differentially expressed genes

Bowtie 0.12.7 was used to map unique consensus sequence tags (a total of two or more reads from all libraries) to the *P. patens* reference genome. Bowtie is an ultrafast, memory-efficient short-read aligner [31]. Bowtie indexes the genome with a Burrows-Wheeler index to keep its memory footprint small. This method performs effectively with DGEP data sets, which are reduced in size and complexity since reads are collapsed to unique tags before mapping. Finally, a preprocessed database of all possible CATG+17-nt tag sequences was created using reference gene sequences. All clean tags were mapped to the reference sequences and allowed no more than 1-nt mismatches. Clean tags mapped to reference sequences from multiple genes were filtered (Table 1). For genes that have multitags found in Solexa tags, the sum of all tags was considered as the gene expression value.

**Figure 2:**
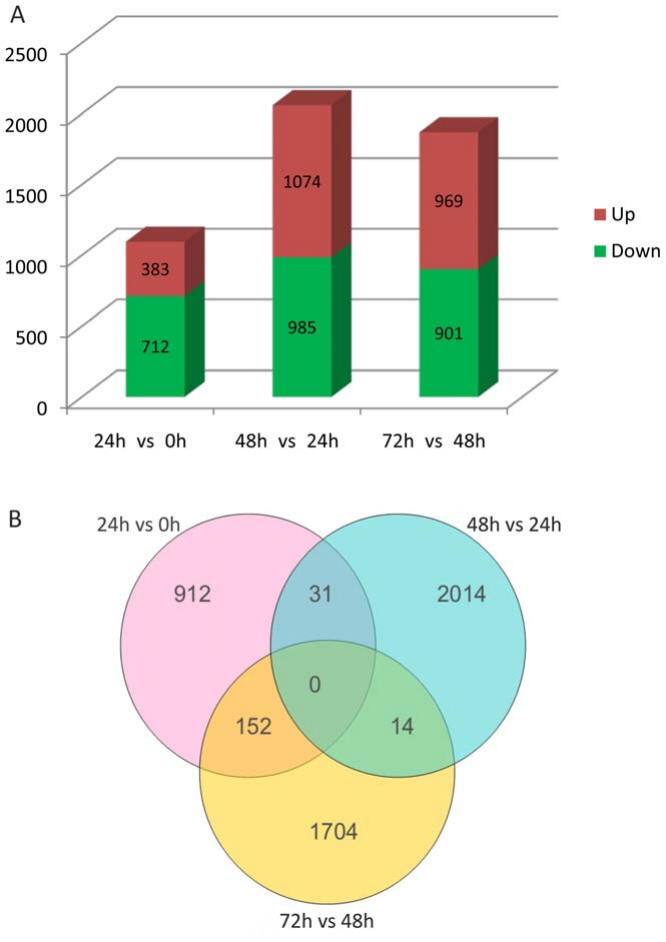
Genes differentially expressed at different time-points during protoplast reprogramming into stem cells. (A) Genes differentially expressed during specific phases of protoplast reprogramming into stem cells were separated into two groups on the basis of whether they were significantly up-regulated or down-regulated. (B) Venn diagrams showing the number of differentially expressed genes during specific time-points of protoplast reprogramming into stem cells.

To compare gene expression profiles, we employed the TMM method from *edgeR* (*empirical analysis of digital gene expression in R*) to normalize the tag distribution per library and determine significance values for differentially expressed genes based on their relative abundance, which reflected the difference in number of tags between each two libraries. The *edgeR* algorithm uses an empirical Bayes approach to improve power in small sample sizes [32]–[34]. This approach accounts for biological and technical variation and has been implemented for tag-based data sets where small numbers of replicates are tested and standard errors disperse further from the mean at low versus high levels of expression [33], [35]. We used *P* values≤0.01, false discovery rates (FDR)≤0.01, and |logFC|≥2 as the threshold to determine differentially expressed genes (DEGs). The transcripts detected in the four libraries are shown in Fig. S3. The red dots were deemed to be differentially expressed transcripts. The black dots represented transcripts that were arbitrarily designated as ‘no difference in expression’ between the two comparative libraries (Fig. S3). As a result, 4827 differences were identified (Fig. 2; Table S1). The full transcriptomic data set was deposited in the GEO database (accession no. GSE36117).

### Quantitative real-time PCR analysis

**Figure 3:**
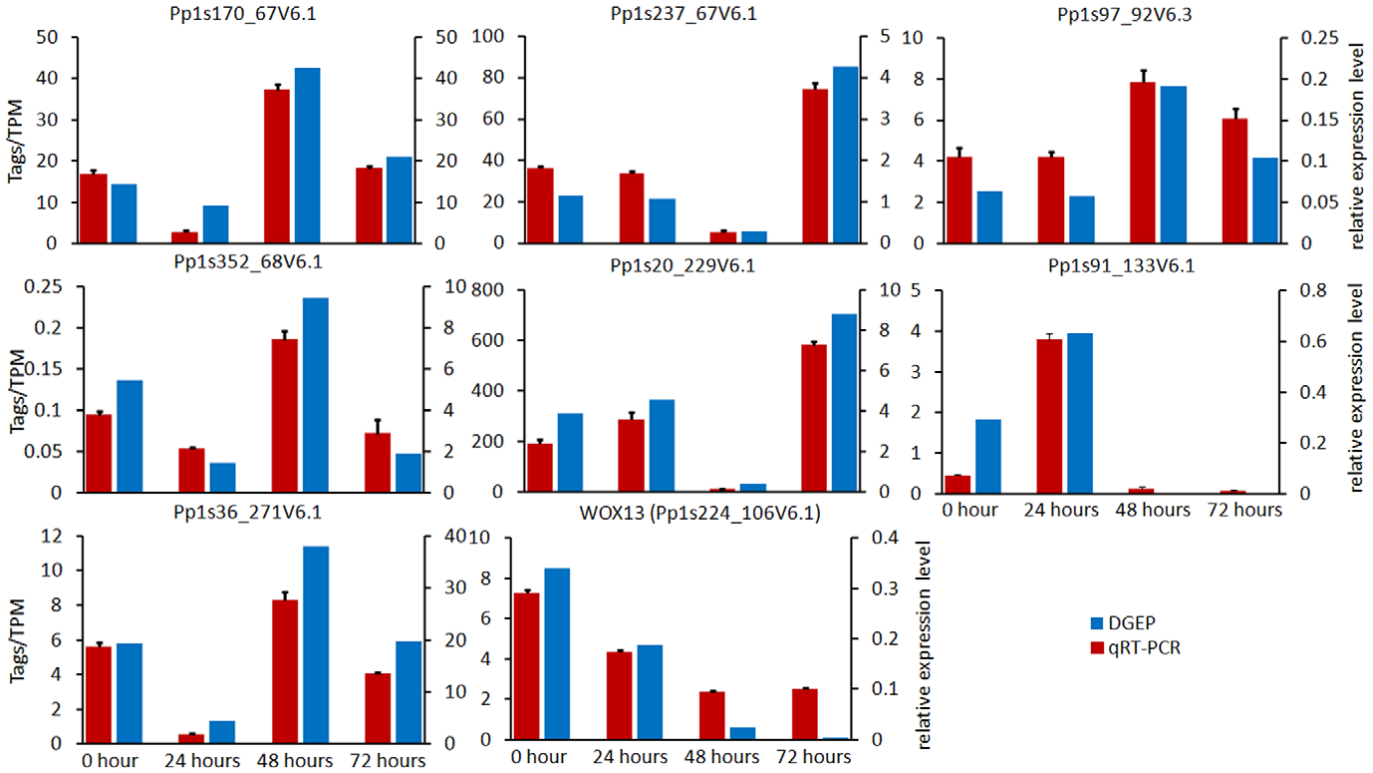
Digital gene expression tag profiling and quantitative real-time PCR analysis of the expression of five randomly selected genes. All real-time PCR reactions were repeated three times and the data are presented as the mean ± SD. The *x*-axis indicates the sampling time-points and cell types. The *y*-axis shows the expression levels: the left bar (red color) shows tag number per million tags by DGEP and the right (blue color) shows the relative expression level by qRT-PCR.

To test the reliability, accuracy and reproducibility of the DGEP data, quantitative real-time PCR analysis was applied to validate the expression pattern of eight randomly selected genes (Table S2). Among these genes, a homologue of the Arabidopsis stem cell maintenance gene *WOX13* (Pp1s224_106V6, which we designated *PpWOX13a*) was included, which exhibited a relatively high expression level in t-0 protoplasts and 24-hour cultures and subsequently was down-regulated. A putative heat shock cognate protein-encoding gene (Pp1s97_279V6), for which expression was relatively stable in all four libraries, was chosen as a reference gene for data normalization. For each gene, the trends in quantitative real-time PCR (qRT-PCR) expression profile were in agreement (i.e., up- or down-regulation) with that obtained by DGEP analysis (Fig. 3, Table S3).

### Global gene expression profiles during protoplast reprogramming

Among all DEGs, 1095 genes showed a change in expression level in t-24 cells compared to t-0 protoplasts, of which 383 genes were up-regulated and 712 genes were down-regulated. A total of 2059 DEGs were detected between culture for 24 h to 48 h and 1074 genes showed transcript accumulation. Of the 1870 DEGs detected between 48 h to 72 h culture, 969 genes were up-regulated and 901 genes were down-regulated (Fig. 2A; Table S1). Among the DEGs, 4630 genes were preferentially expressed in a single comparison, 197 genes were expressed in two comparisons, and none were shared in all three comparisons (Fig. 2B).

To understand further details of the DEGs, significant enriched gene ontology (GO) terms were identified according to their *P* value and enrichment factor. The top 15 significant enriched GO terms in each group are summarized in Table S4 and Fig. S4. The DEGs were significantly enriched in the processes of a variety of biotic and abiotic responses (including responses to salt stress, cold, cadmium ions and bacterial infection), photosynthesis-related processes, glycolysis, ribosome biosynthesis and translation. However, the number, expression level and description of DEGs differed at different stages during protoplast reprogramming. These results indicated that these biological processes played important roles in protoplast reprogramming. The expression changes of DEGs involved in photosystem II repair and responses to blue light, high light intensity and heat occurred within the first 24 h of protoplast reprogramming. In addition, alteration in expression of DEGs involved in thylakoid membrane organization, protein importation into chloroplast stroma, photorespiration, chloroplast organization, and response to abscisic acid (ABA) might also be necessary for stem cells to re-enter the cell cycle. Similarly, a requirement for stem-cell division and differentiation was reflected in expression of DEGs involved in purine nucleotide biosynthesis, embryo development, fatty acid biosynthesis, protein folding, photoinhibition and response to wounding. Significantly enriched molecular functions of DEGs were 3-chloroallylaldehyde dehydrogenase activity, poly (U) RNA binding, chlorophyll binding, structural constituent of ribosome, copper ion binding and catalytic activity. Some DEGs that functioned in glyceraldehydes-3-phosphate dehydrogenase activity, *p*-*p*-bond-hydrolysis-driven protein factor activity, translation initiation factor activity binding and rRNA binding were significantly enriched from 24 and 72 h. These results indicated DEGs that functioned in binding, protein synthesis and catalytic activity were important during protoplast reprogramming. Furthermore, DEGs that functioned in phosphoglycerate kinase activity, aminomethyltransferase activity, acetyl-coA carboxylase activity, glutamate-ammonia ligase activity, oxidoreductase activity that acted on paired donors, glutathione binding, ATPase activity coupled to transmembrane movement of substances and calmodulin binding (from 0 to 24 h), alpha-amylase activity, NADH dehydrogenase (ubiquinone) activity and GTP binding (from 24 to 48 h), and phosphoglucomutase activity, ubiquitin binding, protein histidine kinase activity and cobalt ion binding (from 48 to 72 h), might also be vital for the transition from protoplasts to stem cells and stem-cell division and differentiation.

The cellular components of DEGs were mainly enriched in the chloroplast stroma, chloroplast thylakoid, and chloroplast envelope, followed by the vacuolar membrane, ribosome, stromule and apoplast, which indicated that the molecular biological reactions mainly occurred in the chloroplast, vacuolar membrane, ribosome, stromule and apoplast. In addition, some DEGs functioned in mitochondria from 24 to 72 h. Few DEGs that functioned in the cell wall were specifically expressed.

**Figure 4:**
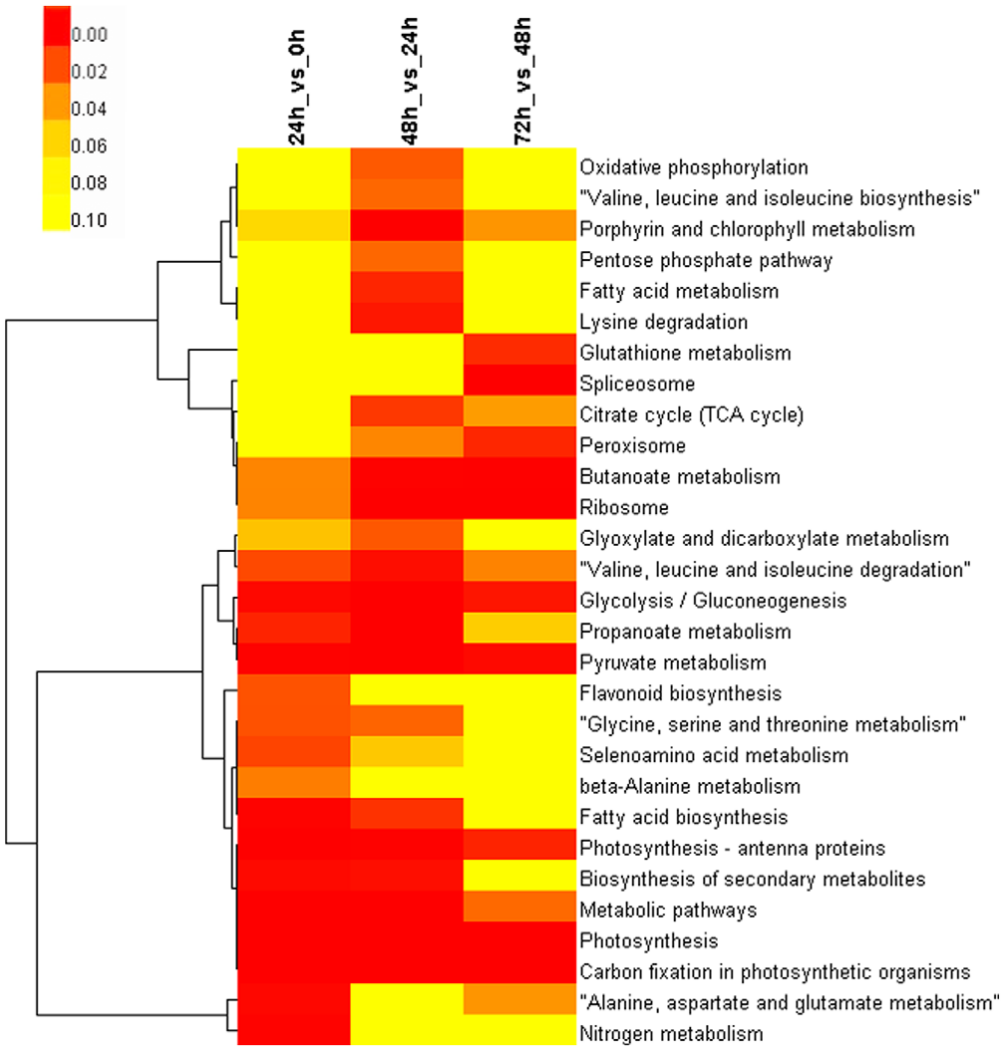
Heatmap of significantly enriched pathways. The yellow and red color shows the p-value of significantly enriched pathways.

**Figure 5:**
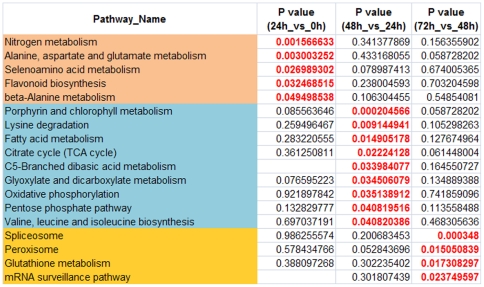
Specific enriched pathways at specific stages during protoplast reprogramming into stem cells.

To investigate the functions of DEGs during protoplast reprogramming, significant enriched Kyoto Encyclopedia of Genes and Genomes (KEGG) pathways were identified according to the *P* value and enrichment factor. The top 15 significant enriched KEGG pathways in each comparison are summarized in Table S5 and Fig. S5. Hierarchical clustering of significant pathways showed that the photosynthesis, butanoate metabolism, ribosome, glycolysis/gluconeogenesis and pyruvate metabolism pathways were involved in all processes of protoplast reprogramming (Fig. 4), which indicated that these pathways are essential for cell survival, division and differentiation. Some genes involved in the nitrogen metabolism, alanine, aspartate and glutamate metabolism and selenoamino acid metabolism pathways were specifically enriched within the first 24 h of protoplast reprogramming, early stage of stem cell reprogramming. Porphyrin and chlorophyll metabolism, lysine degradation, fatty acid metabolism, citrate cycle (TCA cycle), C5-branched dibasic acid metabolism, glyoxylate and dicarboxylate metabolism, oxidative phosphorylation, and pentose phosphate pathways and valine, leucine and isoleucine biosynthesis were specifically enriched from 24 to 48 h, a stage of stem cell re-entering cell cycle. Thus these pathways were indicated to be closely associated with the cell fate transition during protoplast reprogramming into stem cells. Meanwhile, spliceosome, peroxisome, glutathione metabolism and mRNA surveillance pathways were specifically enriched from 48 to 72 h (Figs. 4–5), which indicated that these pathways were closely related with the protonemal regeneration after stem cell reprogrammed.

### Key candidate factors involved in protoplast reprogramming

**Figure 6:**
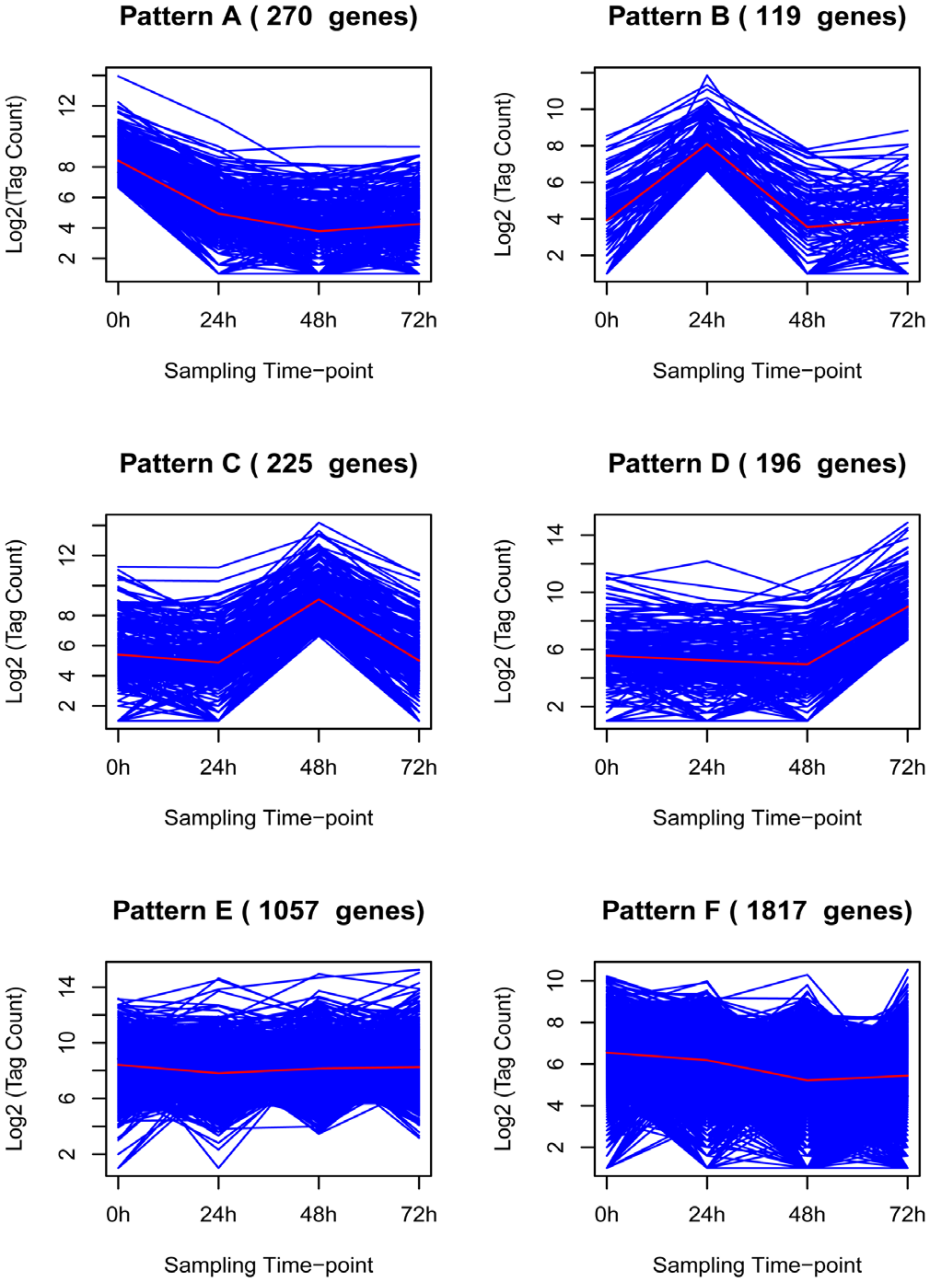
Patterns of gene expression by K-means cluster analysis in the developing gametophyte of *P. patens*. Differentially expressed genes across all four time-points were grouped into six patterns using the K-means clustering algorithm. The *y*-axis gives the tag count (on a log_2_ scale) of differentially expressed genes. Each line represents a different gene.

To elucidate the molecular controls of protoplast reprogramming into stem cells in *P. patens*, we further transformed the comparison of two sequential time-points into a comparison with the expression level in t-0 protoplasts as a common reference. Based on the transformed comparison data and on the basis of mRNA accumulation trends, we performed a K-means clustering analysis using MEV, which partitioned the 4827 DEGs into six clusters (Fig. 6) [36]. The gene number in each pattern ranged from 119 (pattern B) to 1817 (pattern F). Patterns A to D contained genes preferentially expressed in fresh protoplasts (pattern A), cells that had regenerated a new cell wall (pattern B), reprogrammed stem cells (pattern C) and regenerated 2- to 3-celled chloronemata (pattern D) (Fig. 6).

Compared with cells of six-day-old protonemata, freshly isolated protoplasts differed in shape and gene expression because of removal of cell walls. Most of the 270 preferentially expressed genes in fresh protoplasts functioned in material synthesis and degradation, energy supply and abiotic stress responses (Figs. 1C and 6; Table S6). In addition, more than 20 transcription factors, 16 kinase activity-related genes, a number of phytohormone metabolism and signaling-related genes, and three methyltransferase-encoding genes also showed peak expression level in freshly isolated protoplasts. It is interesting that several homologues of stem cell-associated transcription factors, such as NAC2, CUC2, RD26 and WOX13, in vascular plants demonstrated maximal expression levels.

One hundred and nineteen genes in pattern B showed peak expression levels in t-24 cells, which represented a key stage in the reprogramming of *P. patens* protoplasts (Figs. 1D and 6; Table S5). Among the annotated genes, most were related to material and energy metabolism, including a few genes involved in amino acid metabolism and starch synthesis and genes encoding organic materials degradation (Fig. 4; Table S5). Less than 15 genes that encoded transcription factors were specifically expressed. In addition, five methylation-associated genes and two chromatin remodeling genes also exhibited significantly high expression levels.

Genes in pattern C were specifically expressed at 48 h, at which time the reprogrammed protoplasts re-entered the cell cycle (Figs. 1E and 6; Table S5). In contrast to fresh protoplasts and t-24 cells, genes involved in photosynthesis and structural constituents of the ribosome were the most abundant categories at this time-point, followed by energy metabolism and fatty acid biosynthesis genes. Some DEGs were related to phytohormone signaling transduction. As for regulatory factors, four transcription factors were maximally expressed, including a putative bZIP1, KAN2 and HRD.

Pattern D contained the genes preferentially expressed in cells cultured for 72 h, at which point stem cell reprogramming was complete and chloronemata regeneration had been initiated (Figs. 1F and 4; Table S5). Of the 196 genes preferentially expressed, the majority functioned in metabolism and protein synthesis (Table S5). A few genes that encoded putative transcription factors and phytohormone signal transduction-associated factors were also included in this pattern. In addition, a putative gene (root hair specific 8; *RHS8*) that involved in root hair development in vascular plants [37] was specifically expressed. According to previous reports, protonemal apical cells in mosses, like root hairs in flowering plants, are tip-growing cells, and some genes involved in plant cell tip growth were also functionally conserved between *Physcomitrella* and flowering plants [38], [39] Thus, we speculated that *RHS8* might play an important role during protonema/chloronema development after stem cell reprogrammed in *P. patens*. In addition, a putative cyclin-dependent protein kinase, *CAK1AT*, which encoded a homologue of *CDK-activating kinase 1AT* and functioned in maintenance of root meristem identity in Arabidopsis [40], was maximally expressed.

It is well known that protein methylation and phytohormones play vital roles throughout all aspects of plant growth and development, including stress responses. In the present study, several genes that encode homologues of Arabidopsis methyltransferase showed maximal expression within 24 h of protoplast culture (i.e. Patterns C and D; Fig. 4, Table S5). More than 30 phytohormone-related genes, which included almost all phytohormone categories, showed maximal expression in fresh protoplasts. However, a few genes that associated with three main phytohormones, namely auxin, ABA and jasmonic acid, showed dynamic expression levels throughout the protoplast reprogramming process. From comparison of the gene number and category at each stage, we suggest that phytohormones might play important roles in the early stage of protoplast reprogramming into stem cells.

## Discussion

### *Physcomitrella patens* is an excellent system for exploration of the mechanisms of protoplast reprogramming into stem cells

At recent annual moss meetings (2007) and the 21^st^ International Conference on Arabidopsis Research in 2010, the *P. patens* apical stem cell system received considerable attention. In addition to the advantages of the system already outlined, cell divisions can be followed easily in time and space because nearly all gametophytic cells are in direct contact with the environment (i.e., there are no ‘hidden’ cell layers). In addition, the control of cell fate over time and space can be studied both in the protonemal cell lineage and in the more complex tissue of the gametophore. Orthologues for many genes are present in angiosperms and close paralogs are present in gymnosperms. The genome of *P. patens* encodes homologues of stem cell-related genes, such as three WOX paralogs and NAC transcription factors [16], [41]. However, how these genes function during development or protoplast regeneration is still unclear. The morphological and structural characteristics allow us to track the mechanisms both at the single-cell level and in living plants. Compared to other protoplast systems, *P. patens* protoplasts are readily isolated from young protonemal tissue [2]. A major advantage of the *P. patens* system is the ease with which cells can be reprogrammed into pluripotent stem cells. Another advantage of the *P. patens* ‘protoplast–stem cell system’ is that protoplast regeneration occurs at a high frequency, does not require supplementation of the medium with hormones, and results directly in the formation of chloronemal filaments or even a whole gametophyte and does not undergo a callus stage [24]. In these respects the process thus resembles spore germination [24]. Flow cytometry analysis of the cell cycle also showed high synchronization of the process after protoplast isolation.

Because of the higher degree of synchronization, the ease with which reprogramming to stem cells is induced, and the shorter times for regeneration, the *P. patens* protoplast–stem cell system is a more favorable system with which to explore molecular mechanisms for stem cell reprogramming than other protoplast systems, such as tobacco, cucumber and clover blossom [6], [9], [42].

### Altered metabolism and stress responses are necessary for protoplast survival and reprogramming into stem cells

In the course of protoplast isolation from protonemata, a cell rapidly (within seconds) changes its form and is converted into a geometrically ideal sphere. The communication between cells is interrupted. In order to survive, protoplasts must first cope with the alteration of the surrounding environment and renew the cell configuration. We propose that a cascade of stress signal reactions is generated when a protoplast is completely separated from a cell wall. Such reactions might be similar to those accompanied by plasmolysis of plant cells caused by drought or salt. Changes in the expression levels of stress-response genes in the protoplasts might be partially associated with the process of protoplast separation *per se*. This process is likely to be stressful for a protonemal cell because the cell wall is enzymatically degraded and the protoplast is separated from its mechanically rigid, protective polysaccharide envelope into a hypertonic solution. The process of protoplast separation from a cell wall is a type of cell wounding. To survive, the protoplast must initiate the repair mechanism to confront the damage induced by cell wall removal, which represent alteration of metabolism of other materials and energy supply. Our results show that the significantly enriched pathways, which were concentrated in decreased photosynthesis, increased fatty acid, O-glycan, flavonoid, flavone and flavonol biosynthesis, and propanoate metabolism, contribute to the survival and rejuvenation of the protoplast.

Results from morphogenetic observation and FCM (Fig. 1) show that after culture for 48 h most protoplast-derived cells have been successfully reprogrammed into stem cells and re-enter the cell cycle. In order to satisfy the need for rapid division and growth, the cells show enhanced photosynthesis and protein synthesis as well as metabolism of other materials and energy supply.

After 72 h culture, the reprogrammed stem cells had divided once or twice, and chloronema development was initiated. The characteristics of metabolic processes were similar to those at 48 h. The increased expression levels of several embryogenesis-related genes indicated that protoplast-derived chloronema development showed similarities to embryo development.

### Key candidate factors and regulatory mechanisms involved in protoplast reprogramming into stem cells in *P. patens*

To maintain the stem cell state, it is important that multiple regulation factors are coordinated, such as transcription factors, phytohormone synthesis and signaling transduction, and DNA and protein modification, except for alterations in metabolism.

### 1. Stem cell-related genes and their roles

For survival, the protoplast must also cope with induction of reprogramming into stem cells, therefore activation of genes associated with stem cell sustenance and meristem identity is essential. Our results showed that a number of homologues of meristem identity-related transcription factors, including NAC2, CUC2, RD26, WOX13 and BAM2, and one putative protein kinase exhibit maximal expression levels in freshly isolated protoplasts (Table S6). NAC2, CUC2 and RD26 encode transcriptional activators of the NAC gene family in Arabidopsis [43]. NAC2 is known to control age-dependent senescence and salt-promoted senescence [44]. RD26 is induced in response to desiccation, is localized to the nucleus and acts as a transcriptional activator in the ABA-mediated dehydration response [45]. The higher expression level of these genes may be correlated to the alteration of the culture medium, which contained a higher concentration of mannitol after removal of the cell wall. CUC2 expression in the leaf sinus region is required for serration and the extent of serration is modulated by mir164A-mediated repression of CUC2 [46], [47].

It was interesting that a homologue of *WOX13*, a member of the Wuschel-related homeobox (*WOX*) gene family in vascular plants, showed peak expression in freshly isolated protoplasts and a relatively high level of transcription was maintained after 24 h culture (Fig. 3, Tables S1 and S5). The *WOX* gene family belongs to the homeodomain-containing transcription factors, which are key regulators implicated in the determination of cell fate and cell differentiation in plants [48], [49]. *WOX* genes are specifically expressed in different plant organs and cell types [49]–[54]. *WUS* and *WOX5* genes are two vital members that function in stem cell maintenance in a restricted region of the shoot apical meristem and root apical meristem, respectively [55]–[57]. *WOX13* is among the most highly conserved *WOX* genes and affects root and flower development in Arabidopsis [16]. In *P. patens*, no homologues of *WUS* and *WOX5* are known; of three WOX genes, two are homologues of *WOX13* and one is a homologue of *WOX14* [16]. Therefore, we propose that *WOX* genes in *P. patens* possess broader functions. Our results indicate that the homologue of *WOX13* may play a key role in stem cell identity during protoplast reprogramming into stem cells. Down-regulation of *PpWOX13a* in cells after culture for 24 h may be associated with the smaller apical stem number that led to cell division or the other two WOX homologues may have complementary functions. This finding provides an important clue for further functional study of *WOX* genes in *P. patens* and might also contribute to elucidation of the mechanisms involved in protoplast reprogramming and maintenance of a single apical stem cell in the protonema and leafy shoot apex.

Pp1s352_22V6 encodes a homologue of an Arabidopsis *CLAVATA1*-related receptor kinase-like protein, Barely Any Meristem 2 (BAM2), which is a member of the leucine-rich receptor-like protein kinase family that is required for both shoot and flower meristem function in Arabidopsis [58]. This gene shows a broad expression pattern and is involved in multiple developmental processes, such as vascular strand development in the leaf, control of leaf shape, size and symmetry, male gametophyte (especially anther) development, and ovule specification and function [55], [56]. In addition, the BAM2 expression pattern supports both an early role in promoting somatic cell fates and a subsequent function in pollen mother cells.

In Arabidopsis, stem cell identity maintenance is dependent on the WUS-CLV feedback regulation loop in the shoot apical meristem or on WOX5-SCR/SHR feedback regulation in root apical meristems, which are both dependent on stem niche [59]. *P. patens* lacks a stem cell niche and only one apical stem cell is present in the protonema tip and leafy shoot apex [16]. In the present study, the higher expression levels of homologues of *WOX13* and *BAM2* indicate that the two genes may play pivotal roles in protoplast transformation into stem cells. We further hypothesize that protoplast reprogramming into stem cells and maintenance of the single stem cell identity may be partially determined by interaction of WOX and BAM proteins. However, further experiments are needed to test this hypothesis.

### 2. Epigenetic/methylation modification of protein during stem cell reprogramming

Genetic evidence indicates that, similar to animals, stem cells in plants possess a specialized chromatin structure. This may reflect their capacity for a variety of gene-expression programs, as well as their ability to divide repeatedly without either differentiation or senescence. In recent years, epigenetic mechanisms that control chromatin structure and function, including DNA methylation and histone modification, have emerged as key factors in the regulation of cell growth and differentiation and, thereby, the nuclear reprogramming necessary for dedifferentiation [60]. Protein methylation is one type of protein post-translational modification and has been most studied for histones, which can act epigenetically to repress or activate gene expression [61], [62].

Several genes that encode homologues of methyltransferase in Arabidopsis, including Pp1s233_104V6 (protein arginine methyltransferase 4B, PRMT4B), Pp1s271_68V6 (cobalamin-independent synthase family protein, ATMS1), Pp1s31_85V6 (protein-l-isoaspartate methyltransferase 1, PIMT1), Pp1s35_262V6 (protein-l-isoaspartate methyltransferase 2, PIMT2) and Pp1s8_46V6 (homocysteine methyltransferase 2, HMT2), are preferentially expressed within 24 h of protoplast isolation, a stage of stem cell reformation. These results indicate that protein methylation may be an additional important epigenetic mechanism for establishment and/or maintenance of the undifferentiated state during protoplast reprogramming into stem cells.

### 3. Endogenous phytohormones and phytohormone-responsive genes

How the regulators described above actually control the behavior of protoplast reprogramming into stem cells in *P. patens* is still largely unknown. Phytohormones are the most likely candidates as regulators of developmental switches, and it has been proposed that hormones play a central role in mediating the signal transduction cascade that leads to the reprogramming of gene expression. Our results indicate that the requirement for a wide variety of endogenous phytohormones during protoplast reprogramming into stem cells is largely determined by the stage of the cultures.

Cytokinins have many roles in plant development, one of which is to stimulate cell division in the shoot in vascular plants [63]. Cytokinins induce bud formation in mosses in a concentration-dependent manner [64]. Cytokinin biosynthesis and signal transduction might be necessary for the activation of protoplast reprogramming processes, but not for subsequent stem cell reprogramming processes. For example, isopentenyl transferase 9 (IPT9), which catalyzes the first step in isopentenyl-type cytokinin (a major cytokinin in moss) *de novo* biosynthesis [65], shows maximal expression in freshly isolated protoplasts.

Moreover, several ethylene-response genes show preferential expression during both stages of initiation of reprogramming and chloronemal cell division and differentiation after stem cell formation. However, some genes related to the synthesis and signaling of auxin, ABA and jasmonic acid are also altered the transcriptional levels throughout the reprogramming process after removal of the cell wall. Thus, we speculate that a dynamic balance in the interactions among phytohormones, especially of auxin, ABA and jasmonic acid may be pivotal in distinct stages of protoplast reprogramming and switching cell fate during protoplast reprogramming into stem cells, through coordinated interactions with many metabolic pathways (for example, photosynthesis, cell respiration, and protein synthesis and degradation). Nevertheless, further experiments are necessary to verify this conclusion.

### Conclusion

In this study, we analyzed the transcriptome during *P. patens* protoplast reprogramming into stem cells. The transcript levels of 4827 genes were significantly increased or reduced, of which the majority changed only during a specific stage. Our results provide an extensive catalogue of regulatory factors and related genes involved in protoplast survival, reprogramming, and cell division during protoplast reprogramming into stem cells in *P. patens*. Potential applications of these data include identification of candidate genes and as targets for reverse genetic studies of stem cell maintenance and its evolution, and as tools for exploration of the molecular mechanisms underlying stem cell reprogramming in plants.

## Materials and Methods

### Preparation of protoplasts and culture conditions

Protonemal tissue of the Gransden 2004 wild-type strain of *Physcomitrella patens* subsp. *patens* was subcultured at weekly intervals on solid BCDAG medium overlaid on cellophane and containing 5 mM ammonium tartrate [66]. Protoplasts were isolated from six-day-old protonemal tissues following the modified protocol of Grimsley et al. [67]. Ten Petri dishes contained preplasmolyzed protonemal filaments pretreated with 0.48 M mannitol supplemented with 10 ml of 1% solution of Driselase in 0.48 M mannitol (Sigma-Aldrich, USA). After agitated incubation for 30 min in the dark at 25°C, the culture was successively passed through sieves of pore size 100 µm and 50 µm. The filtrate was incubated without agitation under the same conditions for 15 min. Then the protoplast suspension was precipitated by centrifugation for 5 min at 600 rpm and the pellet was washed twice with 0.48 M mannitol by centrifugation at the same rate. The number of protoplasts was determined with a hemocytometer. A portion of the freshly isolated protoplasts were used immediately for FCM analysis and RNA isolation. The remainder of the protoplasts were cultured in BCDAG liquid medium without hormones, but supplemented with 5 mM ammonium tartrate and 0.48 M mannitol, and cultured in the dark for 24 h at 25±1°C. Then the cultures were incubated under a 16/8 h (light/dark) photoperiod regime with light intensity at the surface of the vessels of 55 µmol m^−2^ s^−1^ provided by Philips TLD25 fluorescent lamps.

### Flow cytometry analysis of DNA content

To analyze the DNA content and cell cycle alteration during protoplast culture, FCM analysis was performed according to the method of Ulrich and Ulrich [68]. Suspensions of intact nuclei were prepared from six-day-old protonema (control), freshly isolated protoplasts (t-0) and cultures incubated for 24 h (t-24), 48 h (t-48) and 72 h (t-72) after protoplast isolation by chopping the material with a razor blade in a glass Petri dish that contained 500 µl specific buffer (45 mM MgCl_2_•6H_2_O, 30 mM sodium citrate, 20 mM MOPS, 0.2 mg/ml Triton X-100) [69]. Nuclei from mature Arabidopsis leaves and rice root tips were prepared for determination of the cell cycle stage of the protoplast and its derived cultures. The solution was filtered twice through a sieve of 30 µm pore size. After filtration, a final concentration of 2 µg/ml DAPI (Sigma-Aldrich, USA) was added and incubated for at least 5 min in the dark to stain the nuclei. FCM analysis was conducted with a Becton Dickinson FACSort system (Becton Dickinson, Mountain View, CA, USA). Data were analyzed using Quanta SC software.

### Preparation of digital expression libraries

To achieve comprehensive gene expression profiling during protoplast reprogramming in *P. patens*, samples from t-0 protoplasts and t-24, t-48 and t-72 cultured cells were pooled for RNA isolation and library construction. Total RNA was isolated using TRIzol reagent (Invitrogen, Carlsbad, CA, USA) following the manufacturer's instructions. RNase-free DNase (RQ1; Promega Corporation, USA) was used to remove genomic DNA contamination. RNA was quantified using a NanoDrop ND-1000 spectrophotometer (Thermo Fisher Scientific, USA). RNA quality was tested at an absorbance of A_260_/A_280_>2.0 with an Agilent 2100 Bioanalyzer using the RNA 6000 LabChip Kit (Agilent Technologies, Palo Alto, CA). Only RNA with RNA integrity values greater than 8.0 were used for digital gene expression tag profiling (DGEP).

Tag libraries for each mRNA 3′ terminus were prepared with the Digital Gene Expression Tag Profiling Kit version 2.1B (Illumina, San Diego, CA, USA) in accordance with the manufacturer's protocol. Six micrograms of total RNA was extracted and mRNA was purified using biotin-Oligo (dT) magnetic bead adsorption. Messenger RNA enriched on the beads by oligo (dT) was converted into double-stranded cDNA through reverse transcription. While on the beads, double-strand cDNA was ligated with *Nla*III endonuclease to produce a bead-bound cDNA fragment that comprised the sequence from the 3′-most CATG to the poly (A)-tail. Magnetic bead precipitation was used to purify the cDNA fragments with 3′ ends and add the Illumina adapter 1 to their new 5′ ends. The junction of the Illumina adapter 1 and the CATG site was recognized by *Mme*I, which is a type I endonuclease with separate recognition sites and digestion sites. *Mme*I cuts 17 bp downstream of the CATG site to produce 17 bp cDNA sequence tags with adapter 1. After removing 3′ fragments by magnetic bead precipitation, the Illumina adapter 2 was ligated to the 3′ end of the cDNA tags, which generated tags with different adapters at both ends to form a tag library.

### Digital gene expression tag profiling

Linear PCR amplification with 15 cycles was performed with primers complementary to the adapter sequences to enrich the samples for the desired fragments. The 85 bp fragments were purified by 6% TBE PAGE gel electrophoresis. These fragments were then digested and the single-chain molecules fixed onto a Solexa Sequencing Chip (flowcell) for Solexa sequencing by an Illumina Cluster Station and Illumina Genome Analyzer II System (version 1.0). Each molecule grows into a single-molecule cluster sequencing template by *in situ* amplification. Four types of nucleotides, which were labeled by different colors, were added and sequencing by synthesis was performed. Each tunnel generated millions of raw reads with a sequence length of 35 bp.

### Sequence annotation and identification of differentially expressed genes

Raw reads were transformed into clean tags by trimming adaptor-only tags, filtering low-quality tags (those that contained ambiguous bases), tags that were too long (>21 nt) or too short (<21 nt), and tags that consisted of a single copy (probably the result of sequencing error). The remaining tags were designated distinct clean tags. The genomic sequences corresponding to the tags were retrieved from the *Physcomitrella* genome sequence assembly (ftp://ftp.jgi-psf.org/pub/JGI_data/phytozome/v7.0/Ppatens/annotation/). All distinct clean tags were aligned to the reference *P. patens* database with Bowtie 0.12.7 (http://bowtie-bio.sourceforge.net/) and tolerances were set to allow no more than 1 nt mismatch. Tags that mapped multigenes were removed. The number of distinct clean tags for each gene was calculated and then normalized using the TMM method in *edgeR* (http://www.bioconductor.org/packages/2.3/bioc/html/). Saturation analysis was performed to check whether the number of detected genes continued to increase as the total tag number increases. Gene models for all of the up- and down-regulated tags in this study can be obtained from the *Physcomitrella* Genome Browser using the gene ID number assigned to each tag feature.

A rigorous algorithm to identify DEGs between two samples was developed for significance testing [70]. The *P* value corresponds to the differential gene expression test. The FDR was applied to determine the threshold *P* value in multiple tests and analyses [71]. The number of tags mapped to a reference gene was considered as the expression abundance of the gene. Expression abundances of a gene from two samples were compared to determine differences in expression. A gene was considered to show a statistically significant change in expression between samples when the expression difference was more than four-fold the cutoff value (|log_2_Ratio|≥2) with *P* value≤0.01 and FDR<0.01.

### Quantitative real-time PCR assay

Samples were prepared using the method described above and total RNA was isolated from three biological repeats of t-0 protoplasts and t-24, t-48 and t-72 cultured cells. DNA contamination was removed from each RNA sample using RQ1 RNase-free DNase (RQ1; Promega Corporation, USA). cDNA synthesis was performed with SuperScript™ III reverse transcriptase (Invitrogen) and 5 µg total RNA for each sample using oligo (dT18) primers. For real-time PCR, gene-specific primers were designed using Primer 5 software (Primer 5, Applied Biosystems, USA), assessed by Oligo 6 and synthesized by Shenggong Cooperation (Shenggong, Shanghai, China). Primers used in the real-time PCR assay are listed in Table S2. Real-time PCR was performed using a Corbett Research Rotor-Gene 3000 under the following conditions: 94°C for 5 min (1 cycle); 94°C for 20 s, 50–60°C for 20 s, and 72°C for 20 s (50 cycles). Transcript abundances were identified using the SYBR Green PCR Master Mix (TaKaRa). Each reaction contained 1× mix buffer, 0.25 µM each primer, and about 2 ng cDNA in a final volume of 20 µl. qRT-PCR for each gene was performed on three biological replicates with three technical replicates per biological replicate. Melting curves were performed on the product to test if only a single product was amplified without primer–dimers and other bands. The resulting products with all primer combinations were visualized on a 2% agarose gel to confirm the generation of a single product of the correct size. Pp1s97_279V6 expression was used as an internal control to normalize all data. Relative quantitative analysis was performed using comparative quantitation with Rotor-Gene Real-Time Analysis Software 6.1. Significant variation from the internal control was determined using Student's *t*-test where *p*≤0.05 was considered to be differentially expressed.

### Gene Ontology functional and pathway enrichment analysis of differentially expressed genes

Gene Ontology (GO, http://www.geneontology.org/) is an international standardized gene functional classification system that offers a dynamic-updated controlled vocabulary and a strictly defined concept to comprehensively describe properties of genes and their products in any organism. GO covers three domains: cellular component, molecular function, and biological process. GO functional enrichment analysis was used to identify significant enriched GO terms in DEGs. Statistical significance of GO terms was calculated with the following formula [36]:
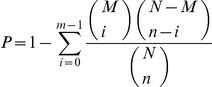
where *N* is the number of all genes with GO annotation, *n* is the number of DEGs in *N*, *M* is the number of all genes that are annotated to certain GO terms, and *m* is the number of DEGs in M. GO terms with a *P* value<0.05 were significantly enriched in DEGs. The top 15 significantly enriched GO terms in each DEG set were considered for further analysis on the basis of the enrichment factor.

Pathway enrichment analysis was used to identify significantly enriched metabolic pathways or signal transduction pathways in DEGs compared with the whole genome background. Pathway analysis of DEGs was performed using the KEGG database and Fisher's exact test algorithm to calculate the statistical significance of each pathway [36]. The formula used was the same as that for GO analysis. In this instance, *N* is the number of all genes with KEGG annotation, *n* is the number of DEGs in *N*, *M* is the number of all genes annotated to specific pathways, and *m* is number of DEGs in *M*. Since a large number of pathways were involved, we implemented FDR correction to control the overall Type I error rate of multiple hypotheses. The *Q* value was calculated to determine the threshold *P* value in multiple tests and analyses [72]. Pathways with a *Q* value<0.05 were significantly enriched in DEGs. In addition, a cluster analysis was performed using the statistical significance pathway data of the three sample groups with Cluster 3.0 (http://smd.stanford.edu/resources/restech.shtml). The *x*-axis was the pathway's *p* value, and the *y*-axis was the different sample groups [73].

### Identification of key candidate factors during protoplast reprogramming

To identify the key candidate factors during protoplast reprogramming, the comparison of two consecutive time-points was transformed into a comparison using the t-0 time-point as a common reference point. We obtained the union of the three DEG groups, and performed a K-means clustering analysis with MEV (http://www.tm4.org/mev/) using the normalized gene expression level of each DEG.

## Supporting Information

Figure S1 **Distribution of distinct clean tags in each sample.**
(TIF)

Figure S2 **Saturation evaluation of expression of unique tags in each sample.**
(TIF)

Figure S3 **Smear plots from the** ***edgeR*** **-based analysis of gene expression.** Genes are plotted based on their log-fold change of transcript abundance between two compared samples on the *y*-axis and log concentration on the *x*-axis for raw tag libraries separately. Differentially expressed genes are shown in red.
(TIF)

Figure S4 **Top 15 significantly enriched GO terms in each sample.**
(TIF)

Figure S5 **Top 15 significantly enriched pathways in each sample.**
(TIF)

Table S1 **List of differentially expressed genes during protoplast reprogramming into stem cells.**
(XLS)

Table S2 **Primer sequences of the selected and internal genes used for real-time PCR analysis.**
(DOC)

Table S3 **List of the** ***R^2^*** **value between DGEP and qRT-PCR.**
(XLS)

Table S4 **Significantly enriched GO terms of differentially expressed genes.**
(XLS)

Table S5 **Significantly enriched pathways.**
(XLS)

Table S6 **Complete gene list of each K-means cluster during protoplast reprogramming in** ***P. patens*** **.**
(XLS)
