# Supplementary figures and images for: Transcriptome of Protoplasts Reprogrammed into Stem Cells in Physcomitrella patens
Source: PLoS One. 2012 Apr 24;7(4):e35961. doi: 10.1371/journal.pone.0035961 (PMC3335808; doi:10.1371/journal.pone.0035961)

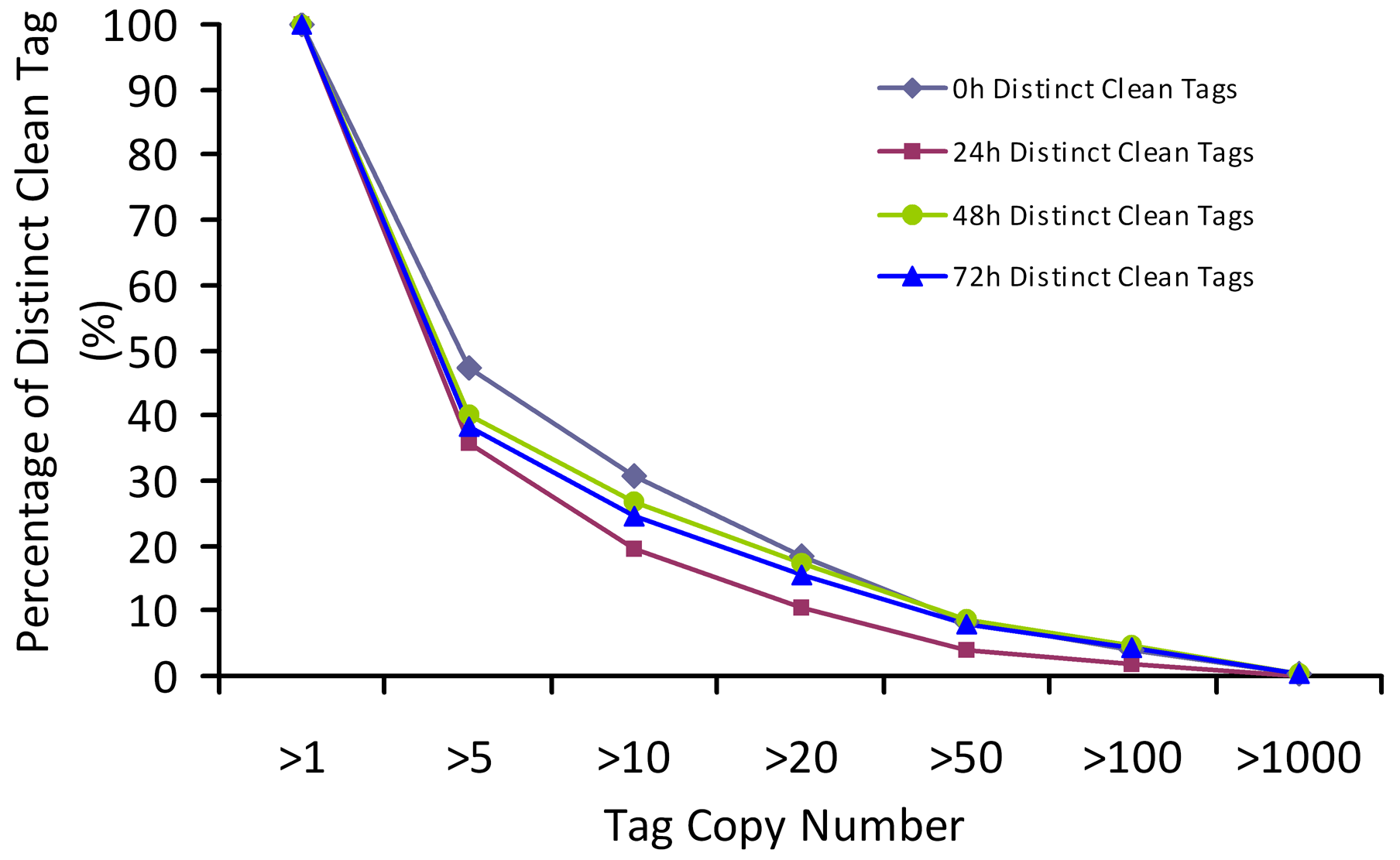

Supplement: Figure S1 — Distribution of distinct clean tags in each sample. (TIF) [file pone.0035961.s001.tif]

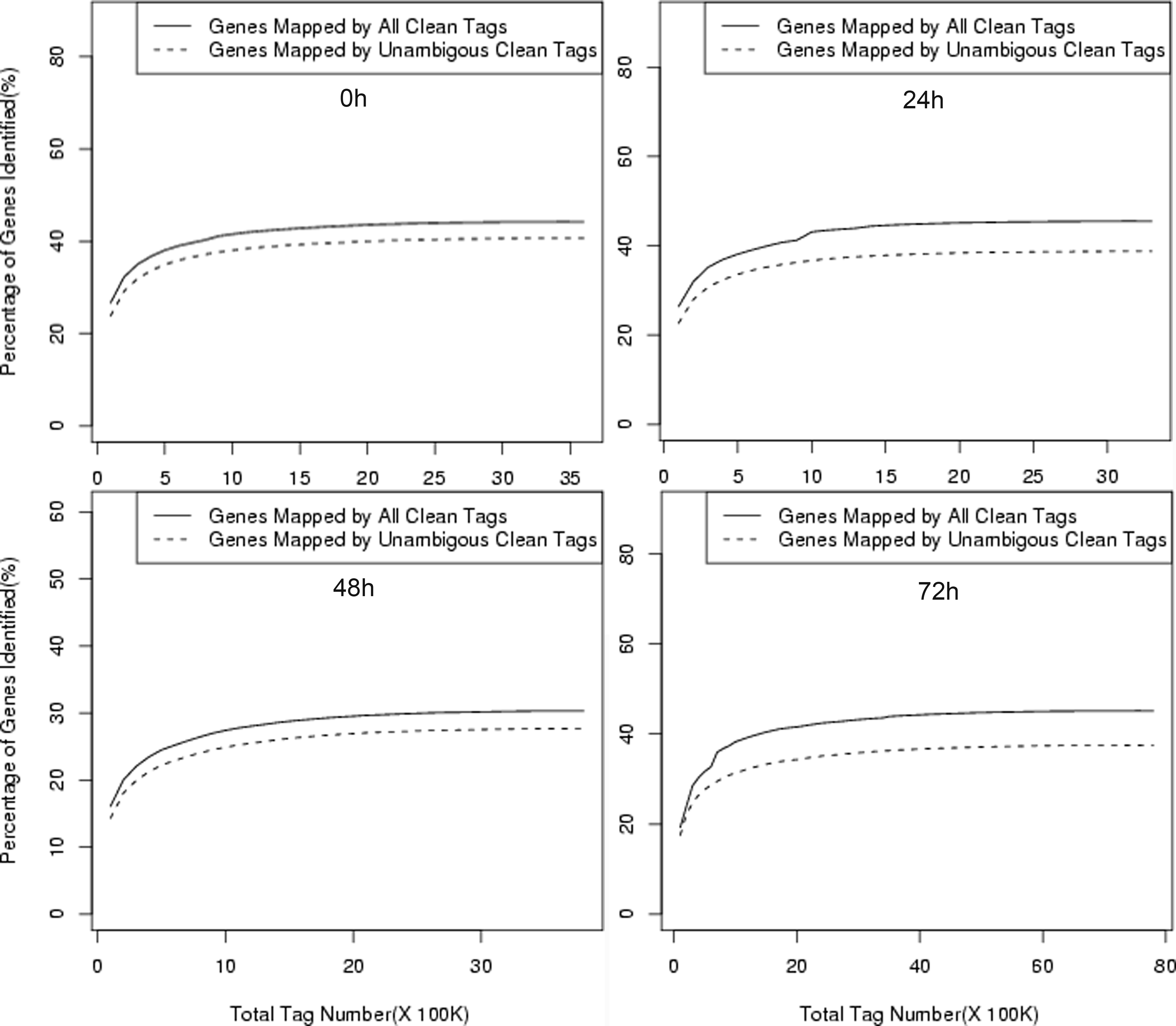

Supplement: Figure S2 — Saturation evaluation of expression of unique tags in each sample. (TIF) [file pone.0035961.s002.tif]

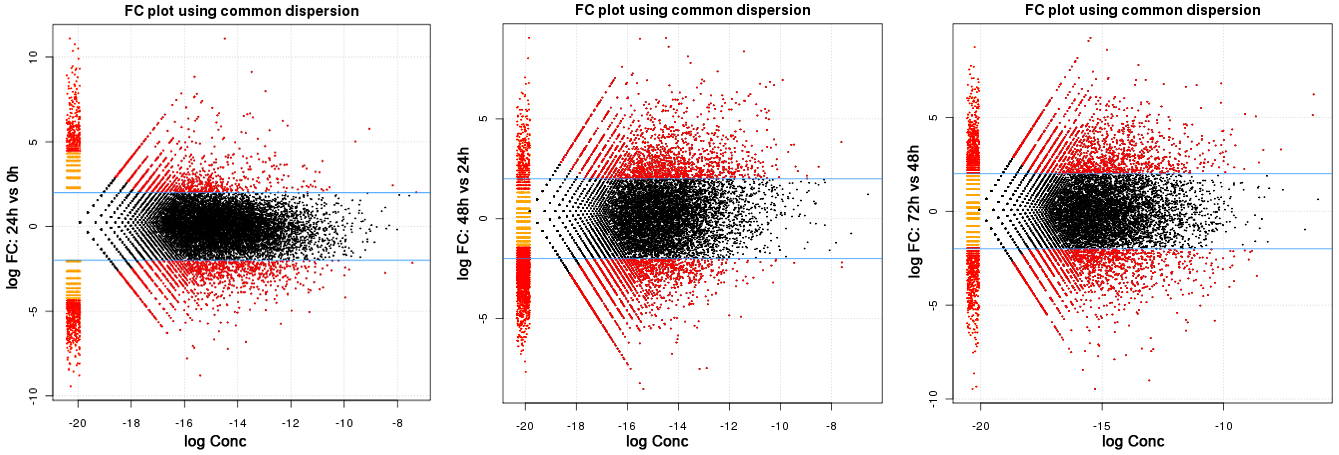

Supplement: Figure S3 — Smear plots from the edgeR -based analysis of gene expression. Genes are plotted based on their log-fold change of transcript abundance between two compared samples on the y-axis and log concentration on the x-axis for raw tag libraries separately. Differentially expressed genes are shown in red. (TIF) [file pone.0035961.s003.tif]

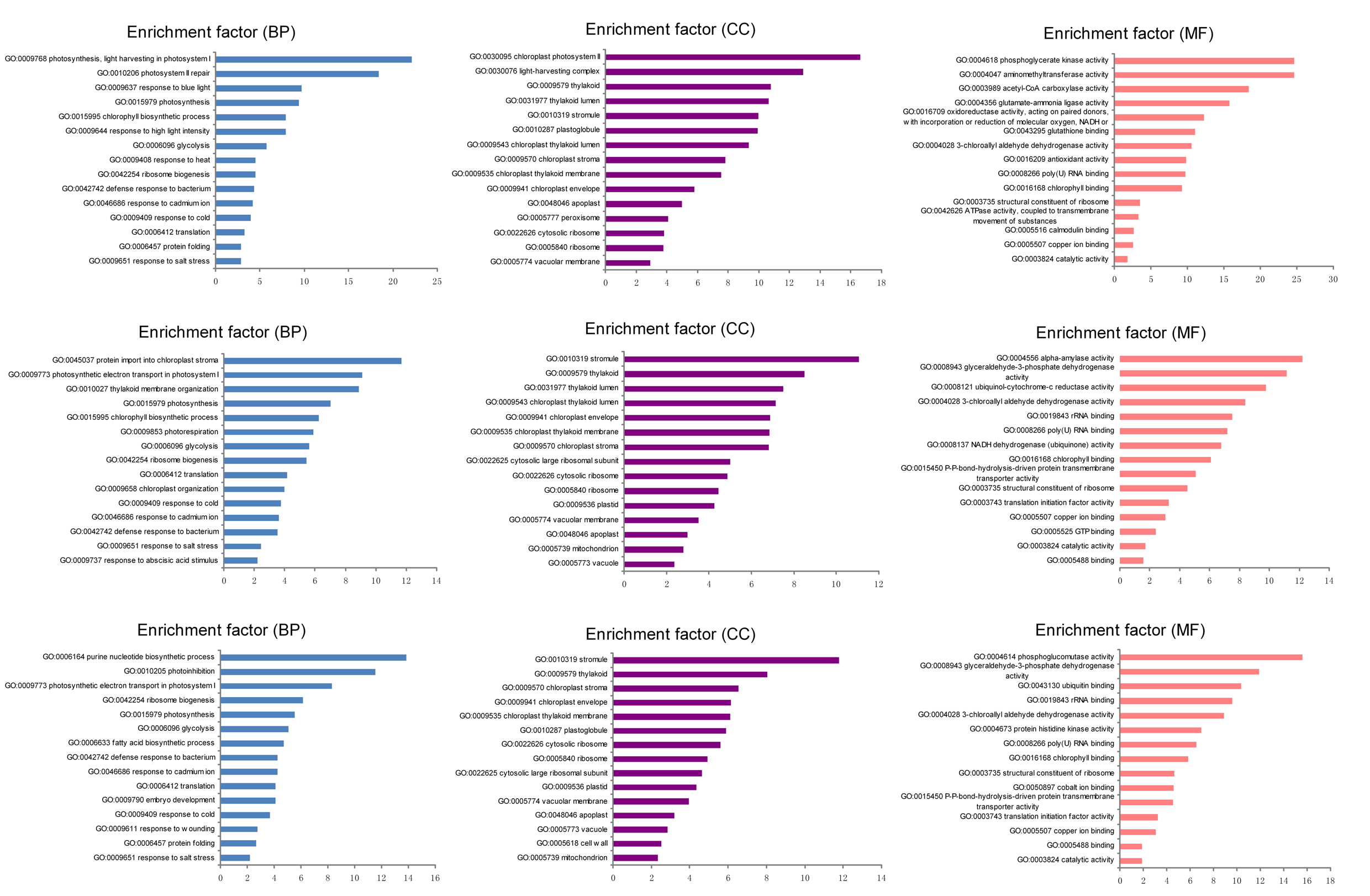

Supplement: Figure S4 — Top 15 significantly enriched GO terms in each sample. (TIF) [file pone.0035961.s004.tif]

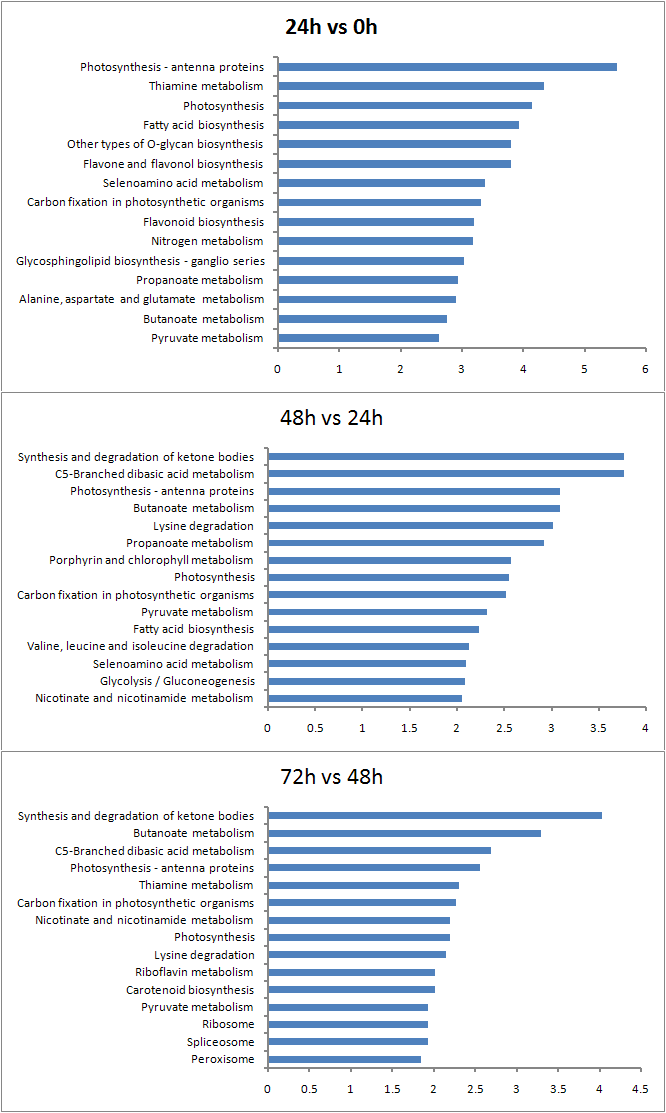

Supplement: Figure S5 — Top 15 significantly enriched pathways in each sample. (TIF) [file pone.0035961.s005.tif]
