# Supplementary material for: Transcriptome of Protoplasts Reprogrammed into Stem Cells in Physcomitrella patens
Source: PLoS One. 2012 Apr 24;7(4):e35961. doi: 10.1371/journal.pone.0035961 (PMC3335808; doi:10.1371/journal.pone.0035961)
Supplement: Table S2 — Primer sequences of the selected and internal genes used for real-time PCR analysis. (DOC) [file pone.0035961.s007.doc]

Table S2. The primer sequences of the selected genes for quantitative Real-Time PCR.

| Protein ID | Gene symbol | sense primer | reverse primer |
| --- | --- | --- | --- |
| Pp1s170_67V6.1 | EMB1467 | GGGAGGGAGTGATGGAGT | TGCTGTGAATGCGTATGG |
| Pp1s237_67V6.1 | TOM3 | GTGGGAGGAGGAGTTGG | TCTCAGTTCTATCCGTATCAGT |
| Pp1s97_92V6.3 | MAPR3 | GGACGAGTGATGCTTTGT | TTTGGGAACGATAGTGAGA |
| Pp1s352_68V6.1 | LPD1 | AGACTAAGGAGCCGAAGG | TGGCGTCACCAATACAAT |
| Pp1s20_229V6.1 | MEE58 | GCGTCTGATGAACCTGGGATG | AGTGGGCGGGCTTGTATGG |
| Pp1s91_133V6.1 | KT1 | TTCTTGTTCGCACCCAT | AGTTCGTTGAAGTGACCC |
| Pp1s36_271V6.1 | CKS2 | CGCTCAGCCTCGTCTTC | TCCGATACCACGCCATT |
| Pp1s224_106V6.1 | WOX13 | CAACAAACAGCGCATTAAA | ACATGAGGTCCCGAGTG |
| *Pp1s97_279V6.1 |  | AGTTATGTCGCATTCACCG | GCCTTCCAATCAGCCTTT |

* Internal control: HSC70-1
